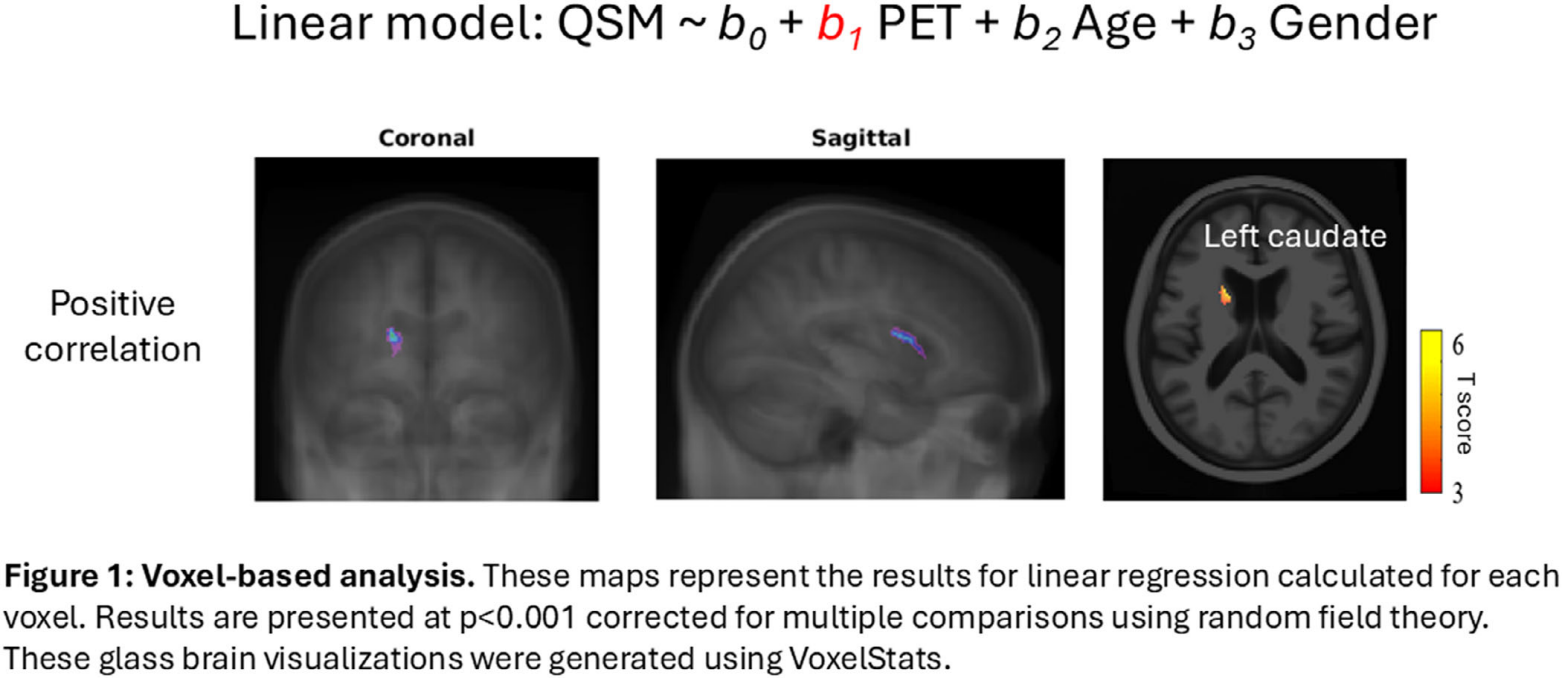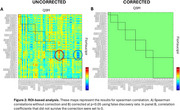# Relationship between AV‐1451‐PET and quantitative susceptibility mapping in atypical Alzheimer's disease

**DOI:** 10.1002/alz70856_099937

**Published:** 2025-12-25

**Authors:** Neha Singh‐Reilly, Ryota Satoh, Jonathan Graff‐Radford, Mary M. Machulda, Val J Lowe, Keith A. Josephs, Jennifer L. Whitwell

**Affiliations:** ^1^ Mayo Clinic, Rochester, MN, USA; ^2^ Department of Neurology, Mayo Clinic, Rochester, MN, USA; ^3^ Department of Psychiatry and Psychology, Mayo Clinic, Rochester, MN, USA

## Abstract

**Background:**

Quantitative susceptibility mapping (QSM) can detect iron deposition or myelin loss in the brain by estimating magnetic susceptibility properties. Iron is an important component in neurofibrillary tangles and is known to co‐localize in regions with tangle burden in Alzheimer's disease (AD). Abnormal magnetic susceptibility measured on QSM has been observed in atypical AD, although it is unclear if susceptibility is regionally related to AV‐1451‐PET uptake in atypical AD.

**Method:**

Forty atypical AD patients (visual variant=24, language variant=7, others=9) were recruited by the Neurodegenerative Research group from the Department of Neurology, Mayo Clinic, Rochester, MN. They underwent a 3T MRI scan with a five‐echo gradient echo sequence for calculation of QSM (*n* = 80), Aβ (Pittsburgh Compound‐B) and a ^18^F‐ AV‐1451 PET scan (*n* = 73). All patients showed evidence of Aβ positivity on PET. To assess the relationship between QSM and AV‐1451‐PET, two approaches were used i) Voxel‐based regression analysis using VoxelStats for the whole brain, and ii) region‐of‐interest (ROI)‐based Spearman's correlation analyses using cortical and subcortical ROIs. All models were adjusted for age and sex as covariates and were corrected for multiple comparisons, specifically the VoxelStats regression maps were corrected using random field theory and the ROI‐based correlation plots were corrected using false discovery rate.

**Result:**

At the voxel‐level, positive correlations between AV‐1451‐PET and QSM were only observed in the left caudate (Figure 1). At the ROI‐level, no significant associations were found, although uncorrected data showed a positive association in the occipital lobe (red circle) and a negative association between substantia nigra susceptibility and occipital AV‐1451 uptake (blue circle) (Figure 2). Since these findings did not survive corrections, they should be interpreted cautiously.

**Conclusion:**

Our data provides little evidence that regional AV‐1451 PET uptake is related to susceptibility changes in patients with atypical AD, suggesting that iron deposition may not be associated with regional tau accumulation in AD.